# Dibromidobis{1-[4-(pyridin-4-yl)phen­yl]ethanone-κ*N*}mercury(II)

**DOI:** 10.1107/S1600536811049993

**Published:** 2011-11-30

**Authors:** Wen-Shen Zhang, Zhi-Juan Liu, Feng Xu, Qi-Ning Xun

**Affiliations:** a Shandong Nonmetallic Material Institute, Jinan 250031, People’s Republic of China

## Abstract

In the title compound, [HgBr_2_(C_13_H_11_NO)_2_], the Hg^II^ atom adopts a four-coordinated HgN_2_Br_2_ geometry, formed by two pyridine N atoms from two ligands and two bromide anions. The complex is located on a twofold axis. The coordination geometry is close to forming a see-saw (SS-4) polyhedron, the symmetry-related organic ligands being almost perpendicular; the dihedral angles between the two pyridine rings and between the two benzene rings are 85.5 (4) and 87.7 (4)°, respectively. Within the organic ligand, the pyridine ring is nearly coplanar with the benzene ring [dihedral angle = 13.1 (8)°]. In the crystal, the mol­ecular complexes are connected through weak inter­molecular C—H⋯Br contacts.

## Related literature

For applications of coordination complexes bearing asymmetric ligands, see: Allendorf *et al.* (2009 ▶); Evans & Lin (2002 ▶); He *et al.* (2006 ▶); Hou *et al.* (2010 ▶). For examples of ligands based on a pyridyl ring, see: Fujita *et al.* (2005 ▶); Song *et al.* (2010 ▶).
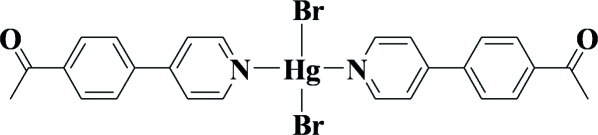


## Experimental

### 

#### Crystal data

[HgBr_2_(C_13_H_11_NO)_2_]
*M* *_r_* = 754.87
Monoclinic, 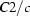

*a* = 16.656 (6) Å
*b* = 5.296 (2) Å
*c* = 29.442 (11) Å
β = 102.453 (6)°
*V* = 2535.8 (16) Å^3^
*Z* = 4
Mo *K*α radiation
μ = 9.25 mm^−1^
*T* = 298 K
0.15 × 0.15 × 0.15 mm

#### Data collection

Bruker SMART APEX diffractometer
Absorption correction: multi-scan (*SADABS*; Bruker, 2003 ▶) *T*_min_ = 0.338, *T*_max_ = 0.338
6208 measured reflections
2358 independent reflections
1339 reflections with *I* > 2σ(*I*)
*R*_int_ = 0.047

#### Refinement

*R*[*F* ^2^ > 2σ(*F* ^2^)] = 0.052
*wR*(*F* ^2^) = 0.131
*S* = 0.99
2358 reflections
151 parameters
25 restraints
H-atom parameters constrained
Δρ_max_ = 1.03 e Å^−3^
Δρ_min_ = −0.41 e Å^−3^

### 

Data collection: *SMART* (Bruker, 2003 ▶); cell refinement: *SAINT* (Bruker, 2003 ▶); data reduction: *SAINT*; program(s) used to solve structure: *SHELXS97* (Sheldrick, 2008 ▶); program(s) used to refine structure: *SHELXL97* (Sheldrick, 2008 ▶); molecular graphics: *SHELXTL* (Sheldrick, 2008 ▶); software used to prepare material for publication: *SHELXTL*.

## Supplementary Material

Crystal structure: contains datablock(s) global, I. DOI: 10.1107/S1600536811049993/bh2387sup1.cif

Structure factors: contains datablock(s) I. DOI: 10.1107/S1600536811049993/bh2387Isup2.hkl

Additional supplementary materials:  crystallographic information; 3D view; checkCIF report

## Figures and Tables

**Table 1:** Hydrogen-bond geometry (Å, °)

*D*—H⋯*A*	*D*—H	H⋯*A*	*D*⋯*A*	*D*—H⋯*A*
C10—H10⋯Br1^i^	0.93	3.01	3.579 (13)	121

Symmetry code: (i) 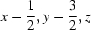.

**Fig. 1.**
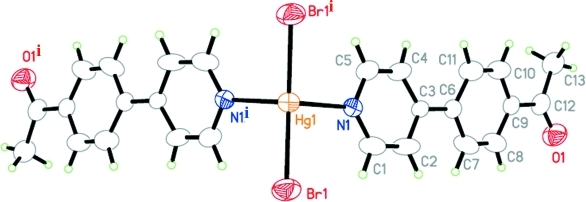
The HgII coordination environment of the title compound, with displacement ellipsoids at the 20% probability level. Unlabeled atoms are generated by symmetry operation -x + 2, y, -z + 1.5.

## supplementary crystallographic information

### Comment

Research on supramolecular compounds from asymmetric organic ligands has become popular because of their potential applications in areas such as magnetic (He *et al.*, 2006), luminescent property (Allendorf *et al.*, 2009; Hou *et al.*, 2010) and nonlinear optical materials (Evans & Lin, 2002). Among available strategies, the geometry of organic ligands is one of the most important factors in determining the structure of the framework. Pyridyl derivatives have been widely used in supramolecular chemistry and many coordination polymers with versatile structures and potential properties have been reported (Fujita *et al.*, 2005; Song *et al.*, 2010). We report herein a molecular complex, Hg*L*_2_Br_2_, generated from an asymmetric organic ligand, 1-(4-(pyridin-4-yl)phenyl)ethanone (*L*) and HgBr_2_.

In the title compound, each Hg^II^ center adopts a distorted HgN_2_Br_2_ tetrahedral coordination geometry, formed by two pyridine N atoms from two ligands and two bromide anions. The N1—Hg1—N1^i^ and Br1—Hg1—Br1^i^ angles are 100.3 (4)° and 147.81 (9)°, respectively [Symmetry code: (i) -*x* + 2, *y*, -*z* + 1.5]. Within the organic ligand, the pyridine ring is nearly coplanar with the benzene ring [dihedral angle: 13.1 (8)°]. In this compound, two ligands *L* are bridged by one Hg^II^ center to form a molecular complex with a see-saw S*S*-4 polyhedron geometry (Fig. 1). The dihedral angles between two pyridyl planes and between two benzene planes are 85.5 (4) and 87.7 (4)°, respectively, close to 90°. So, a feature characteristic of the complex structure is the almost orthogonal arrangement for the two symmetry-related organic ligands.

In the solid state, these molecular complexes associate into a network through weak intermolecular C—H···Br hydrogen bonds, characterized by H···Br, C···Br separations and C—H···Br angle of 3.011 (2), 3.579 (13)Å and 121.0 (7)°, respectively.

### Experimental

A solution of HgBr_2_ (4.7 mg, 0.013 mmol) in CH_3_OH (2 ml) was layered onto a solution of *L* (5.0 mg, 0.025 mmol) in CH_2_Cl_2_ (2 ml). The system was left for about three weeks at room temperature, and colourless crystals were obtained. Yield, 54%.

### Refinement

All non-H atoms were refined with anisotropic displacement parameters. All H atoms were placed in idealized positions and treated as riding to their parent atoms, with C—H = 0.93 (aromatic CH) or 0.96 Å (methyl CH_3_), and *U*_iso_(H) = 1.2 *U*_eq_(carrier C atom), with exception of the methyl H atoms, for which *U*_iso_(H) = 1.5 *U*_eq_(C13). Restraints for anisotropic displacements parameters of C2, C4, C7, C12 and C13 were applied.

### Crystal data

**Table:**

[HgBr_2_(C_13_H_11_NO)_2_]	*F*(000) = 1432
*M**_r_* = 754.87	*D*_x_ = 1.977 Mg m^−^^3^
Monoclinic, *C*2/*c*	Mo *K*α radiation, λ = 0.71073 Å
Hall symbol: -C 2yc	Cell parameters from 1208 reflections
*a* = 16.656 (6) Å	θ = 2.5–18.6°
*b* = 5.296 (2) Å	µ = 9.25 mm^−^^1^
*c* = 29.442 (11) Å	*T* = 298 K
β = 102.453 (6)°	Block, colourless
*V* = 2535.8 (16) Å^3^	0.15 × 0.15 × 0.15 mm
*Z* = 4	

### Data collection

**Table:**

Bruker SMART APEX diffractometer	2358 independent reflections
Radiation source: fine-focus sealed tube	1339 reflections with *I* > 2σ(*I*)
graphite	*R*_int_ = 0.047
φ and ω scans	θ_max_ = 25.5°, θ_min_ = 2.5°
Absorption correction: multi-scan (*SADABS*; Bruker, 2003)	*h* = −17→20
*T*_min_ = 0.338, *T*_max_ = 0.338	*k* = −6→6
6208 measured reflections	*l* = −35→30

### Refinement

**Table:**

Refinement on *F*^2^	Primary atom site location: structure-invariant direct methods
Least-squares matrix: full	Secondary atom site location: difference Fourier map
*R*[*F*^2^ > 2σ(*F*^2^)] = 0.052	Hydrogen site location: inferred from neighbouring sites
*wR*(*F*^2^) = 0.131	H-atom parameters constrained
*S* = 0.99	*w* = 1/[σ^2^(*F*_o_^2^) + (0.0626*P*)^2^] where *P* = (*F*_o_^2^ + 2*F*_c_^2^)/3
2358 reflections	(Δ/σ)_max_ = 0.001
151 parameters	Δρ_max_ = 1.03 e Å^−^^3^
25 restraints	Δρ_min_ = −0.41 e Å^−^^3^
0 constraints	

### Fractional atomic coordinates and isotropic or equivalent isotropic displacement parameters (Å^2^)

**Table:**

	*x*	*y*	*z*	*U*_iso_*/*U*_eq_	
Br1	1.09304 (9)	1.3414 (3)	0.69925 (5)	0.1331 (5)	
C1	0.9296 (7)	0.848 (2)	0.6587 (5)	0.114 (4)	
H1	0.9746	0.9148	0.6488	0.137*	
C2	0.8806 (8)	0.675 (2)	0.6305 (5)	0.115 (4)	
H2	0.8933	0.6333	0.6021	0.138*	
C3	0.8158 (5)	0.5649 (18)	0.6421 (3)	0.069 (2)	
C4	0.8052 (8)	0.640 (3)	0.6837 (5)	0.118 (4)	
H4	0.7634	0.5656	0.6955	0.142*	
C5	0.8535 (8)	0.823 (3)	0.7101 (4)	0.120 (4)	
H5	0.8398	0.8759	0.7376	0.145*	
C6	0.7626 (6)	0.3795 (17)	0.6137 (3)	0.073 (2)	
C7	0.7812 (8)	0.257 (2)	0.5769 (5)	0.123 (4)	
H7	0.8308	0.2937	0.5688	0.147*	
C8	0.7310 (8)	0.081 (3)	0.5511 (5)	0.130 (5)	
H8	0.7470	0.0032	0.5262	0.156*	
C9	0.6581 (6)	0.0203 (17)	0.5617 (3)	0.079 (3)	
C10	0.6395 (8)	0.137 (2)	0.5987 (5)	0.112 (4)	
H10	0.5907	0.0956	0.6075	0.134*	
C11	0.6896 (8)	0.313 (2)	0.6235 (5)	0.118 (4)	
H11	0.6732	0.3910	0.6483	0.142*	
C12	0.6030 (7)	−0.170 (2)	0.5337 (4)	0.103 (3)	
C13	0.5286 (8)	−0.260 (2)	0.5499 (5)	0.122 (4)	
H13A	0.4970	−0.3703	0.5269	0.183*	
H13B	0.4956	−0.1182	0.5546	0.183*	
H13C	0.5455	−0.3508	0.5786	0.183*	
Hg1	1.0000	1.21208 (11)	0.7500	0.0886 (3)	
N1	0.9161 (5)	0.9236 (15)	0.6984 (3)	0.080 (2)	
O1	0.6156 (6)	−0.2498 (18)	0.4976 (4)	0.147 (4)	

### Atomic displacement parameters (Å^2^)

**Table:**

	*U*^11^	*U*^22^	*U*^33^	*U*^12^	*U*^13^	*U*^23^
Br1	0.1385 (11)	0.1556 (13)	0.1188 (11)	−0.0357 (9)	0.0583 (8)	0.0154 (9)
C1	0.106 (9)	0.126 (10)	0.128 (11)	−0.044 (7)	0.062 (8)	−0.044 (8)
C2	0.120 (7)	0.128 (7)	0.113 (7)	−0.019 (6)	0.059 (6)	−0.028 (6)
C3	0.070 (6)	0.082 (6)	0.058 (6)	0.002 (5)	0.021 (5)	0.011 (5)
C4	0.112 (7)	0.150 (8)	0.102 (7)	−0.031 (6)	0.042 (6)	−0.003 (6)
C5	0.108 (9)	0.175 (12)	0.085 (8)	−0.053 (9)	0.035 (7)	−0.031 (8)
C6	0.079 (6)	0.067 (6)	0.071 (6)	0.005 (5)	0.012 (5)	0.003 (5)
C7	0.100 (7)	0.146 (8)	0.130 (8)	−0.023 (6)	0.040 (6)	−0.036 (7)
C8	0.100 (9)	0.166 (12)	0.133 (11)	−0.033 (9)	0.044 (8)	−0.052 (10)
C9	0.086 (7)	0.077 (6)	0.070 (7)	0.000 (5)	0.008 (5)	0.006 (5)
C10	0.109 (9)	0.123 (9)	0.115 (10)	−0.035 (8)	0.049 (8)	−0.006 (8)
C11	0.119 (10)	0.131 (10)	0.113 (10)	−0.045 (8)	0.042 (8)	−0.048 (8)
C12	0.116 (9)	0.101 (8)	0.087 (8)	−0.008 (7)	0.008 (7)	−0.004 (7)
C13	0.130 (10)	0.121 (9)	0.111 (9)	−0.050 (8)	0.019 (7)	−0.006 (7)
Hg1	0.0841 (4)	0.1019 (5)	0.0846 (5)	0.000	0.0283 (3)	0.000
N1	0.071 (5)	0.096 (6)	0.074 (6)	−0.001 (4)	0.016 (4)	−0.007 (5)
O1	0.153 (8)	0.178 (9)	0.110 (7)	−0.043 (6)	0.031 (6)	−0.048 (6)

### Geometric parameters (Å, °)

**Table:**

Br1—Hg1	2.4701 (14)	C8—C9	1.357 (14)
C1—N1	1.301 (13)	C8—H8	0.9300
C1—C2	1.381 (16)	C9—C10	1.346 (14)
C1—H1	0.9300	C9—C12	1.487 (14)
C2—C3	1.335 (13)	C10—C11	1.355 (16)
C2—H2	0.9300	C10—H10	0.9300
C3—C4	1.336 (14)	C11—H11	0.9300
C3—C6	1.459 (12)	C12—O1	1.203 (14)
C4—C5	1.388 (16)	C12—C13	1.498 (17)
C4—H4	0.9300	C13—H13A	0.9600
C5—N1	1.282 (13)	C13—H13B	0.9600
C5—H5	0.9300	C13—H13C	0.9600
C6—C11	1.356 (14)	Hg1—N1	2.383 (8)
C6—C7	1.356 (15)	Hg1—N1^i^	2.383 (8)
C7—C8	1.364 (16)	Hg1—Br1^i^	2.4701 (14)
C7—H7	0.9300		
			
N1—C1—C2	123.4 (10)	C8—C9—C12	120.8 (11)
N1—C1—H1	118.3	C9—C10—C11	122.0 (11)
C2—C1—H1	118.3	C9—C10—H10	119.0
C3—C2—C1	122.8 (11)	C11—C10—H10	119.0
C3—C2—H2	118.6	C10—C11—C6	122.7 (11)
C1—C2—H2	118.6	C10—C11—H11	118.6
C2—C3—C4	112.3 (10)	C6—C11—H11	118.6
C2—C3—C6	124.9 (9)	O1—C12—C9	121.4 (12)
C4—C3—C6	122.7 (9)	O1—C12—C13	118.9 (11)
C3—C4—C5	123.2 (11)	C9—C12—C13	119.6 (11)
C3—C4—H4	118.4	C12—C13—H13A	109.5
C5—C4—H4	118.4	C12—C13—H13B	109.5
N1—C5—C4	123.0 (11)	H13A—C13—H13B	109.5
N1—C5—H5	118.5	C12—C13—H13C	109.5
C4—C5—H5	118.5	H13A—C13—H13C	109.5
C11—C6—C7	114.5 (10)	H13B—C13—H13C	109.5
C11—C6—C3	121.0 (9)	N1—Hg1—N1^i^	100.3 (4)
C7—C6—C3	124.4 (10)	N1—Hg1—Br1	98.7 (2)
C6—C7—C8	123.6 (12)	N1^i^—Hg1—Br1	101.8 (2)
C6—C7—H7	118.2	N1—Hg1—Br1^i^	101.8 (2)
C8—C7—H7	118.2	N1^i^—Hg1—Br1^i^	98.7 (2)
C9—C8—C7	120.4 (12)	Br1—Hg1—Br1^i^	147.81 (9)
C9—C8—H8	119.8	C5—N1—C1	115.1 (9)
C7—C8—H8	119.8	C5—N1—Hg1	119.5 (7)
C10—C9—C8	116.7 (10)	C1—N1—Hg1	125.3 (7)
C10—C9—C12	122.5 (11)		
			
N1—C1—C2—C3	−2(2)	C9—C10—C11—C6	−2(2)
C1—C2—C3—C4	−0.6 (19)	C7—C6—C11—C10	0(2)
C1—C2—C3—C6	−179.5 (12)	C3—C6—C11—C10	−178.3 (12)
C2—C3—C4—C5	4(2)	C10—C9—C12—O1	170.9 (13)
C6—C3—C4—C5	−177.4 (12)	C8—C9—C12—O1	−10.3 (18)
C3—C4—C5—N1	−5(2)	C10—C9—C12—C13	−7.3 (16)
C2—C3—C6—C11	−167.2 (12)	C8—C9—C12—C13	171.5 (12)
C4—C3—C6—C11	14.0 (16)	C4—C5—N1—C1	3(2)
C2—C3—C6—C7	14.3 (16)	C4—C5—N1—Hg1	−175.1 (11)
C4—C3—C6—C7	−164.5 (12)	C2—C1—N1—C5	1(2)
C11—C6—C7—C8	1(2)	C2—C1—N1—Hg1	178.0 (10)
C3—C6—C7—C8	179.1 (12)	N1^i^—Hg1—N1—C5	81.6 (9)
C6—C7—C8—C9	0(2)	Br1—Hg1—N1—C5	−174.7 (9)
C7—C8—C9—C10	−1(2)	Br1^i^—Hg1—N1—C5	−19.6 (9)
C7—C8—C9—C12	179.9 (12)	N1^i^—Hg1—N1—C1	−95.9 (10)
C8—C9—C10—C11	2(2)	Br1—Hg1—N1—C1	7.9 (10)
C12—C9—C10—C11	−179.1 (12)	Br1^i^—Hg1—N1—C1	162.9 (9)

Symmetry codes: (i) −*x*+2, *y*, −*z*+3/2.

### Hydrogen-bond geometry (Å, °)

**Table:**

*D*—H···*A*	*D*—H	H···*A*	*D*···*A*	*D*—H···*A*
C10—H10···Br1^ii^	0.93	3.01	3.579 (13)	121

Symmetry codes: (ii) *x*−1/2, *y*−3/2, *z*.
